# Implications of host sex on liver metabolism during *Schistosoma mansoni* Infection

**DOI:** 10.3389/fcimb.2026.1750683

**Published:** 2026-02-03

**Authors:** Verena von Bülow, Anne Baier, Nicola Buss, Frederik Stettler, Christoph G. Grevelding, Martin Roderfeld, Elke Roeb

**Affiliations:** 1 Department of Gastroenterology, Justus Liebig University Giessen, Giessen, Germany; 2 Institute of Parasitology, BFS, Justus Liebig University Giessen, Giessen, Germany

**Keywords:** autophagy, hepatic metabolism, oxidative stress, *S. mansoni*, schistosomiasis

## Abstract

Various studies suggest that host gender is a disease-determining factor in schistosomiasis. Men have a higher prevalence in endemic areas and show a greater degree of liver damage in chronic infection with *Schistosoma mansoni*. It is currently unclear whether this is only due to socioeconomic causes, such as the working environment and behavior. It was recently suggested that molecular biological differences in the infected host could also contribute to the disease. Current studies in model systems suggest that hormonal influences affect liver metabolism during infection with *S. mansoni*. In particular, a metabolic recycling process in the cell, known as autophagy, could be altered in a sex-specific manner and influence the course of the disease. It is suspected that the differences in metabolism in the liver of *S. mansoni*-infected hosts could contribute to cell stress and thus to organ damage on the molecular level. This article summarizes and discusses known and new aspects of the gender-specific influence on liver metabolism in *S. mansoni* infection.

## Introduction

**Figure 1:**
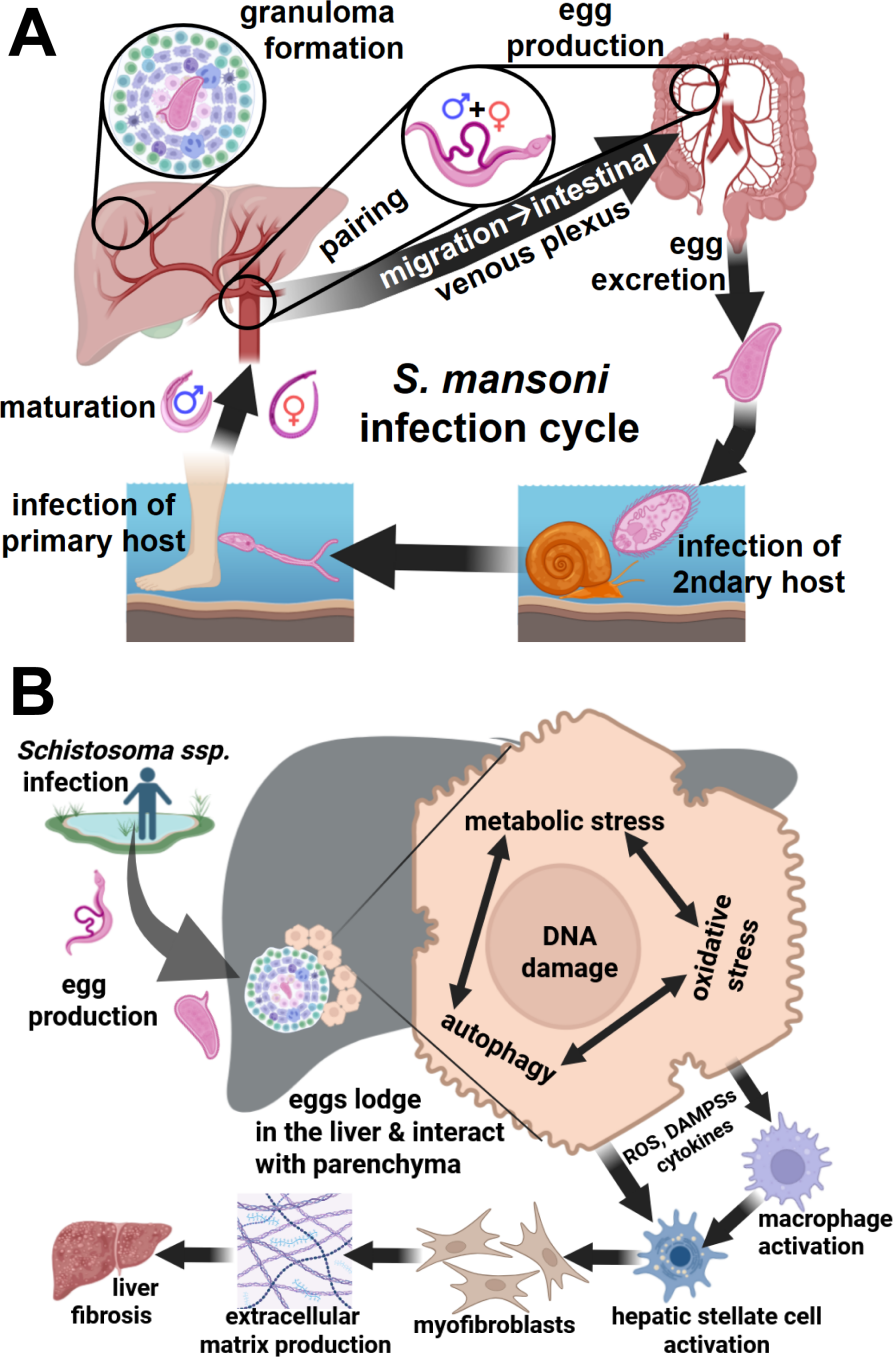
Infecion cycle of *S. mansoni* and metabolic interactions of hepatocytes with trapped *Schistosoma* eggs. **(A)** The *S. mansoni* infection cycle involves the infection of mammals like humans (primary host) and specific freshwater snails (secondary host). Eggs in feces can hatch in fresh water into miracidia, which infect snails. Inside the snails, they reproduce asexually and develop into cercariae. Released cercariae penetrate the skin of the mammalian host, becoming schistosomula, migrating through the bloodstream to the liver, maturing into adult worms, which pair and migrate into mesenteric veins to produce eggs. The eggs can pass the bowel wall and exit humans via feces, restarting the cycle. **(B)** Some eggs can be flushed with the blood stream to other organs where they can be trapped in capilaries due to their size. Eggs that are trapped in smaller branches of the portal vein of the liver can induce an inflammatory response resulting into granuloma formation. The embryos in the eggs can survive for months and mature during this time. The embryos in the eggs reprogram the host´s parenchymal cells, e.g. hepatocytes in the liver, to provide energy and metabolites that are necessary for their development (Bülow et al., 2023). This leads to metabolic exhaustion, cellular stress, autophagy, and finally to DNA damage in hepatocytes. Stress signals first activate macrophages and Kupffer cells, and then hepatic stellate cells, stimulating collagen production. Created with Biorender.

*Schistosoma mansoni* is a parasitic trematode and responsible for schistosomiasis, a neglected tropical disease that affects more than 250 million people worldwide, primarily in sub-Saharan Africa, South America, and parts of the Middle East (Lo et al., 2022). The parasite’s life cycle involves freshwater snails as intermediate hosts and humans as definitive hosts, where it matures in the mesenteric veins and releases eggs that trigger a robust immune response (McManus et al., 2018). The primary pathological consequence of *S. mansoni* infection is hepatointestinal schistosomiasis, characterized by chronic liver inflammation, fibrosis, and metabolic disturbances. Schistosomiasis is transmitted in freshwater environments by schistosome larvae (cercariae) that develop within aquatic snails (**Figure 1A**).

These free-swimming larvae penetrate the skin of individuals who swim or wade in contaminated water. Following skin penetration, cercariae transform into schistosomula, enter the circulation and migrate via the blood and lymphatic vessels to the liver. Within the portal venous system, the parasites mature into adult worms, with male and female worms pairing at this stage. The paired worms subsequently migrate to the venous plexuses of the bladder in *Schistosoma haematobium* infections or to the intestinal venous plexuses in infections caused by *S. mansoni*, *S. japonicum*, and related species. Female worms deposit large numbers of eggs within the small venules of the bladder or intestinal wall, respectively. While a fraction of these eggs traverse surrounding tissues and are excreted—via feces in intestinal schistosomiasis—a substantial proportion become trapped within host tissues, particularly in the portal venous system of the liver. Retained eggs elicit granulomatous inflammation and progressive immune-mediated pathology, ultimately leading to chronic hepatic fibrosis (Schwartz and Fallon, 2018).

The chronic immune response, dominated by a Th2-mediated reaction, leads to fibrosis, hepatomegaly, and portal hypertension (Grzych et al., 1991; Elliott, 1996). Over time, metabolic alterations occur due to the combined effects of inflammation, fibrosis, and changes in immune signaling. Periportal fibrosis is a severe morbidity caused by both current and past exposure to intestinal *schistosomes* (Ewuzie et al., 2025). Recent data obtained from BALB/c mice indicate that activation of the NLRP3 inflammasome in hepatic sinusoidal stellate cells contributes to *Schistosoma japonicum*–induced liver fibrosis through NF-κB–dependent signaling pathways. NLRP3 inflammasome activation occurs in both Kupffer cells (KCs) and hepatic stellate cells (HSCs), with a more pronounced effect observed in KCs. It is hypothesized that inflammasome activation in KCs promotes the production of proinflammatory cytokines, such as interleukin-1β (IL-1β), which subsequently activate HSCs, leading to extracellular matrix deposition and the development of liver fibrosis (Zhang et al., 2019). In addition, patients with schistosomiasis exhibit elevated expression of Hedgehog (Hh) ligands compared with healthy individuals. Activated liver sinusoidal endothelial cells (LSECs) and myofibroblasts are responsive to Hedgehog signaling (Pereira et al., 2013). Soluble egg antigens (SEA) stimulate hepatic macrophages to produce Hh ligands, thereby promoting alternative macrophage activation, fibrogenesis, and vascular remodeling during schistosomiasis (Pereira et al., 2013). Praziquantel (PZQ), the most important drug for the treatment of schistosomiasis, is effective and well tolerated against adult worms of all six *Schistosoma* species that affect humans. However, it does not eliminate the eggs trapped in the liver, nor does it prevent reinfection (Woldegerima et al., 2019), raising concerns about the potential development of drug resistance (Eastham et al., 2024). The liver is a primary target of *S. mansoni* pathology, experiencing profound metabolic disruptions due to chronic inflammation, fibrosis, and oxidative stress. Sex hormones modulate immune responses and liver metabolism, potentially influencing disease progression and severity (Xu et al., 2022; Sciarra et al., 2023). Male and female hosts may metabolize glucose, lipids, and amino acids differently in response to infection, influencing disease outcomes and treatment efficacy. Understanding these metabolic variations can help identifying individuals at higher risk for severe liver fibrosis, which enables earlier interventions. Incorporating sex-specific metabolic data into disease models might even improve predictions of disease progression and treatment responses, aiding clinical decision-making.

Although few studies have been explicitly designed to examine sex as a biological variable in *S. mansoni* infection, this article presents some new aspects and summarizes the available results of studies with male and female hosts to highlight possible gender-specific differences. We recognize the methodological limitations inherent in comparing studies with differing objectives and experimental designs. Therefore, we present such comparisons cautiously and transparently, not as definitive evidence but as hypothesis-generating observations that underscore the need for more targeted research. Excluding these studies altogether would risk overlooking meaningful biological signals that could guide future investigations.

This article focuses on the sex-specific effects of *S. mansoni* infection on liver metabolism. **Figure 1** summarizes the infection cycle of *S. mansoni* and the metabolic interactions of the hepatic parenchyma with trapped *Schistosoma* eggs.

Gender-specific differences in hepatic schistosomiasis are well-documented, with male gender consistently identified as a risk factor for more severe disease progression (Ahmed and Alharbi, 2025; Zongo et al., 2025). While socio-economic, behavioral, and environmental factors have been suggested to contribute to this disparity, emerging evidence indicates that biological and molecular mechanisms play a pivotal role in shaping disease outcomes (Tavakoli Pirzaman et al., 2024). However, the precise molecular pathways underlying these sex-based differences remain largely unexplored.

In general, the risk of hepatocellular carcinoma (HCC) caused by hepatitis B or hepatitis C is significantly higher in men than in women (Yu and Chuang, 2009). However, with increasing age, the effects of alcohol consumption and viral infections on the development of HCC intensify, while the influence of male gender decreases (Yi et al., 2018). Recent findings from meta-analyses, animal models, and *in vitro* studies suggest that *S. mansoni* infection may not only exacerbate liver fibrosis but also predispose individuals to hepatocellular carcinoma (HCC) (Bülow et al., 2021, 2023, 2024). The risk appears to be significantly amplified in the presence of co-infections with hepatitis B virus (HBV) or hepatitis C virus (HCV), which synergistically contribute to chronic liver damage, cirrhosis, and carcinogenesis (Bülow et al., 2021; Allam et al., 2024). In the *S. mansoni-infected* HCC group, the proportion of women was slightly higher at 25% than in the HCC group without schistosomiasis (19%). Due to the low overall number of HCC cases in women, this finding was not statistically significant (Allam et al., 2024). Women are often infected through domestic activities involving contact with water, such as washing clothes and fetching drinking water (Reitzug et al., 2024). In addition, women and people without schooling are among the disadvantaged members of the community who receive little health education and are therefore exposed to an increased risk of *S.mansoni* infection (Adoka et al., 2014). Chronic inflammation driven by *S. mansoni* eggs trapped in hepatic tissue induces a persistent Th2-mediated immune response, oxidative stress, and fibrotic remodeling - all of which are known to create a microenvironment conducive to oncogenic transformation (Härle et al., 2023; Bülow et al., 2024; Russ et al., 2024). Strikingly, gender disparities in HCC incidence among *S. mansoni*-infected individuals mirror global patterns observed in liver cancer, where men exhibit a significantly higher prevalence than women. A recent study reported a male-to-female ratio of 3:1 (75% vs. 25%) among HCC patients with a history of *S. mansoni* infection (Allam et al., 2024). This pronounced inequality justify the assumption that sex hormones, differential immune responses and metabolic differences might influence the course of the disease. Testosterone has been implicated in promoting inflammatory and fibrotic pathways (Ma et al., 2020), whereas estrogen is thought to exert hepatoprotective effects by modulating oxidative stress and inflammatory cytokine production (Xu et al., 2022).

Earlier findings revealed that male mice exhibit lower worm burdens compared to females when infected with *S. mansoni* (Nakazawa et al., 1997). This difference has been linked to the presence of testosterone during early infection stages, suggesting hormonal influences on susceptibility. Testosterone administration to female mice prior to infection reduced worm burden, while castration of male mice increased the corresponding susceptibility, thus highlighting testosterone’s protective role during the early stages of infection (Nakazawa et al., 1997). Female mice infected with *S. mansoni* showed a stronger inflammatory response than male mice (Boissier et al., 2003). This suggests that host sex influences immune defense mechanisms during infection.

Liver fibrosis is a significant pathological consequence of *S. mansoni* infection, leading to severe complications such as portal hypertension and hepatosplenomegaly (Leite et al., 2013). Emerging evidence indicates that male hosts are prone to more severe hepatic fibrosis compared to females, likely driven by sex-specific differences in immune regulation and cytokine expression. A study examined cytokine patterns in schistosomiasis patients with Symmers’ fibrosis (Silva-Teixeira et al., 2004). The findings revealed that male patients with severe fibrosis exhibited significantly higher serum levels of interleukin-4 (IL-4), IL-5, and tumor necrosis factor-alpha (TNF-α) compared to females with similar fibrosis grades. The Th2 cytokines IL-4 and IL-5 as well as TNFα frequently promote fibrogenic signaling pathways indirectly by inducing alternative macrophage activation (M2 macrophages), thereby enhancing the activation and proliferation of hepatic stellate cells (HSCs) and augmenting their collagen synthesis. These cytokines are known to stimulate collagen synthesis, suggesting their involvement in fibrosis development especially in male patients. In our experiments, the two most abundant fibrillar collagens in *S. mansoni*–infected mice were type I and type III collagen, with the highest levels observed in mice infected at 14 weeks of age. Morphometric quantification of the Sirius red-stained areas also showed an accumulation of fibrillary collagen in liver tissue of infected black six mice (Müller et al., 2024). In contrast, female patients with intense fibrosis had lower levels of these profibrotic cytokines, indicating that other mediators may contribute to fibrosis in females (Silva-Teixeira et al., 2004). Epidemiological evidence clearly demonstrates that men exhibit a higher incidence of liver cirrhosis and hepatocellular carcinoma than women (Tapper and Parikh, 2018). A Canadian research group has hypothesized that biological sex influences the responsiveness of hepatic myeloid cells, thereby predisposing men to more severe hepatic inflammation (Kuipery et al., 2022). In particular, hepatic immune cells such as monocytes exhibit an increased pro-inflammatory potential upon activation. This heightened inflammatory response may help explain the elevated risk observed in men for developing liver cirrhosis and, consequently, hepatocellular carcinoma.

## Materials and methods

### Animal experiments

The life-cycle of a Liberian strain of *Schistosoma mansoni* was maintained using *Biomphalaria glabrata* snails as intermediate hosts and Syrian hamsters (*Mesocricetus auratus*) as final hosts, as described previously (Grevelding, 1995; Bülow et al., 2023). Briefly, bisex (bs) and monosex (ms) worm populations were generated via polymiracidial and monomiracidial snail infections, respectively. Hamsters were infected using the paddling method with pre-soaking procedure as described before (Dettman et al., 1989). Bs infections were initiated at 8 weeks of age and maintained for 46 days. Ms infections were carried out with either male or female cercariae and maintained for 67 days to ensure complete maturation of the worms, as female development requires more time in the absence of male partners. The choice of a model system with monosex controls aims to investigate exclusively the effects of eggs on liver metabolism. A monosex control was included to detect worm-related effects, i.e., the effects of the parasite infection. These could potentially mask the effects caused by the eggs. Untreated animals served as supercontrols. All procedures followed ETS No. 123 guidelines and were approved by the Regional Council Giessen (V54–19 c 20/15 c GI 18/10 Nr. A26/2018).

### Western blot analysis

Western blotting was performed as described previously (Roderfeld et al., 2020). Briefly, liver tissue proteins were extracted by homogenization in Lämmli buffer (containing SDS, glycerol, Tris-HCl, bromophenol blue, DTT), denatured at 95 °C for 10 minutes, and separated by SDS-PAGE. Proteins were then transferred onto PVDF membranes, which were blocked and incubated with primary antibodies overnight at 4 °C, followed by HRP-conjugated secondary antibodies. Signal detection was performed using enhanced chemiluminescence (ECL), and band intensities were quantified using ImageJ.

### Malondialdehyde assay

Lipid peroxidation, as an indicator of oxidative stress, was assessed in liver samples using the Malondialdehyde (MDA) Assay Kit (Merck, Darmstadt, Germany) (Bülow et al., 2023), following the manufacturer’s instructions.

### Statistical analysis

The present study is of an exploratory nature. Therefore, the group sizes were not estimated in advance by pre-specified effect sizes. The study was started with an existing number of cryopreserved organs that were not required for the maintenance of the parasite life cycle. Statistical analysis was performed using SPSS version 26.0 (SPSS Inc., IBM corporation, Armonk, NY, RRID: SCR_002865). Subsequent comparison of groups within each Kruskal-Wallis test were Bonferroni-corrected. Because of the exploratory nature of the study, no further adjustment for p values was performed. Densitometrically assessed data from Western blots were presented as mean or median ± 95% confidence intervals (CIs).

## Results and discussion

**Figure 2:**
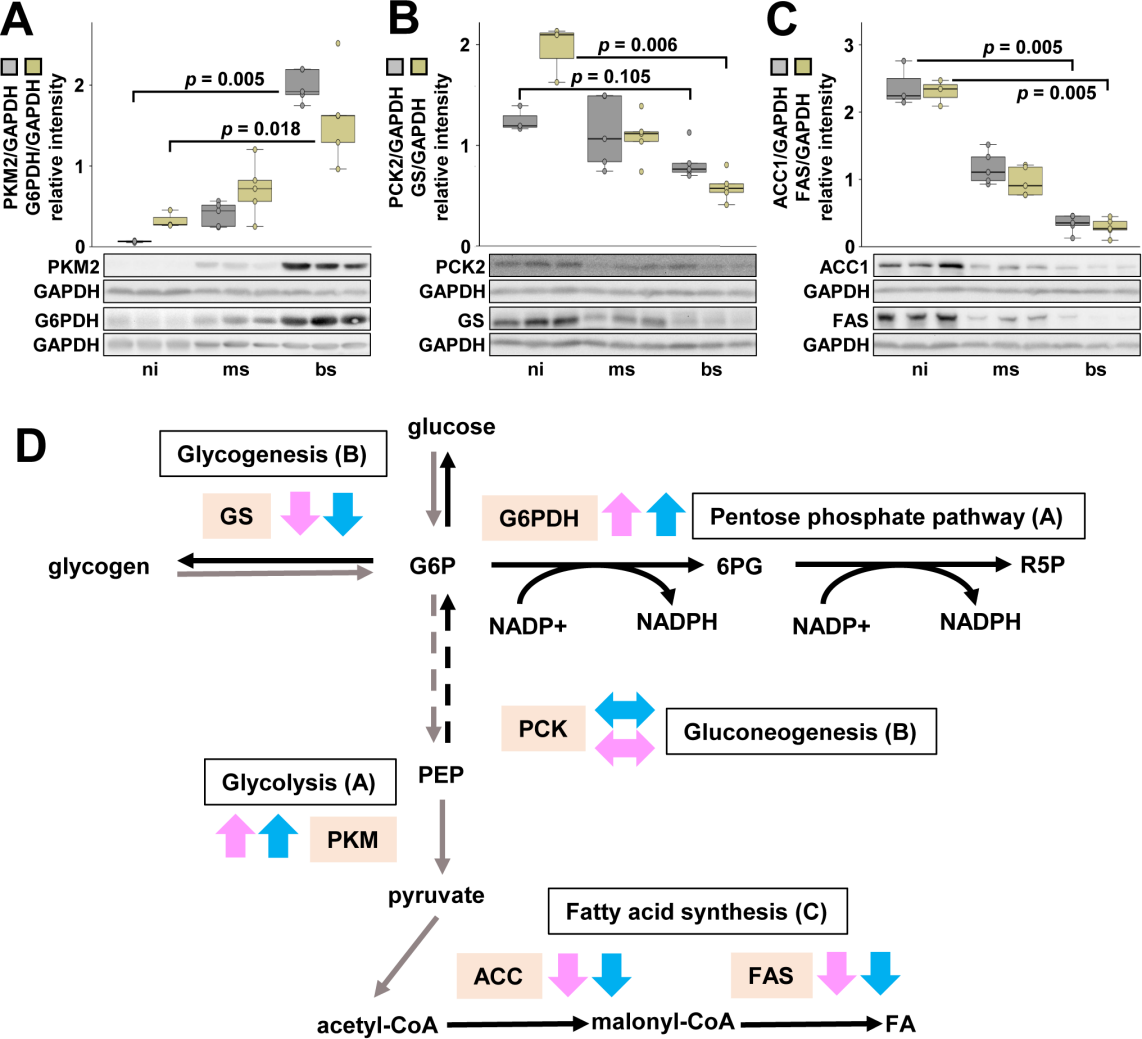
Key enzymes involved in hepatic carbohydrate and lipid metabolism are comparably regulated in male and female hamsters infected with *S. mansoni*. Western blot analysis revealed an upregulation of rate-limiting glycolytic enzymes, including PKM2 and G6PDH **(A)**, along with a downregulation of key enzymes involved in gluconeogenesis (PKC) and glycogenesis (GS) **(B)**, as well as a reduction in lipogenic factors (ACC1 and FAS) **(C)** in the livers of bs-infected male hamsters (n = 3–5). All experiments were independently performed at least three times. Statistical significance was determined using the Kruskal-Wallis test followed by Bonferroni-corrected pairwise comparisons, as indicated in the figure. **(D)** A schematic overview summarizes the regulatory patterns of key enzymes involved in hepatic carbohydrate and lipid metabolism in male and female hosts. PCK, phosphoenolpyruvate carboxykinase, ACC, acetyl-CoA carboxylase; bs, bisex; FAS fatty acid synthase; G6PDH, glucose-6-phosphate dehydrogenase; GCK, glucokinase; GS, glycogen synthase; ms, monosex; ni, non-infected; PKM, pyruvate kinase muscle isozyme; kinase muscle isozyme; PPP, pentose phosphate pathway. Panels A-C depict the regulation of key hepatic enzymes in male hamsters, while the regulation patterns in female littermates were published recently (Bülow et al., 2023). The pink arrows indicate the progression in female animals, while the blue arrows indicate the progression in male organisms.

Deregulation of hepatic carbohydrate and lipid metabolism during Schistosomiasis is independent of host sex The liver is one of the primary organs targeted by *S. mansoni*, where parasite eggs induce chronic inflammation, fibrosis, and metabolic dysfunction. As the infection progresses, these pathological changes disrupt key hepatic processes, including glucose and lipid metabolism, amino acid synthesis, and bile acid homeostasis. These metabolic disturbances are not only central to the disease’s pathogenesis but also contribute substantially to the progression of liver-related complications such as portal hypertension, hepatomegaly, and hepatic fibrosis (Bülow et al., 2023; Buonfrate et al., 2025). A recent study examined the liver proteome of *S. mansoni*-infected hamsters and compared male and female host responses (Ghezellou et al., 2024). Egg deposition by *S. mansoni* was associated with a downregulation of hepatic proteins involved in energy production and metabolic pathways (Ghezellou et al., 2024). Our working group provided detailed information about the regulation of hepatic carbohydrate and lipid metabolism in female hamsters infected with *S. mansoni* (Bülow et al., 2023). Key enzymes of hepatic carbohydrate and lipid metabolism were regulated comparably in female and male hamsters infected with *S. mansoni* (**Figure 2**).

The hepatic expression of pyruvate kinase M2 (PKM2) and glucose-6-phosphate dehydrogenase (G6PD) was significantly elevated in male bisex-infected hamsters (**Figure 2A**), mirroring the regulation seen in female hamsters (Bülow et al., 2023). A similar upregulation of glycolysis-associated genes, including PKM2, has been observed in *Schistosoma japonicum*-infected female mice (Xu et al., 2019). G6PD, crucial for NADPH production, supports fatty acid synthesis and oxidative stress defense (Yang et al., 2019). Given that *S. mansoni* suppresses fatty acid synthesis, increased G6PD may help maintaining NADPH levels for glutathione synthesis (Salvemini et al., 1999), which has been shown to reduce schistosome-induced oxidative stress in hepatocytes (Bülow et al., 2023). Furthermore, glycogen synthase expression was reduced in male bisex-infected hamsters (**Figure 2B**), consistent with female (Bülow et al., 2023) hamsters, while gluconeogenesis enzyme PCK2 expression remained unchanged (**Figure 2B**) (Bülow et al., 2023). Similarly, fatty acid synthase (FAS), acetyl-CoA carboxylase 1 (ACC1), and acetyl-CoA synthetase (ACS) were downregulated in male (**Figure 2C**) and female (Bülow et al., 2023) hamsters, reflecting metabolic suppression seen in *S. japonicum* infection.

*Schistosome* eggs appear to drive a glycolytic metabolic program that enables rapid ATP production in the cytosol under hypoxic conditions, accompanied by a concurrent reduction in oxidative phosphorylation (OXPHOS), the mitochondrial pathway responsible for oxygen-dependent ATP generation. The cells underwent a metabolic reprogramming characterized by a transition from OXPHOS-dependent mitochondrial respiration to a glycolytic metabolic state. These results suggest that schistosome eggs reprogram parts of the host hepatic metabolism by enhancing catabolic pathways such as glycolysis while suppressing anabolic processes such as glycogen synthesis, gluconeogenesis, and fatty acid synthesis. Furthermore, we have previously demonstrated, that *S. mansoni* infection led to hepatic depletion of neutral lipids and glycogen, with schistosome eggs mobilizing and storing host-derived lipids (Bülow et al., 2023). Metabolic disruptions in xenobiotic processing, energy metabolism, and lipid homeostasis were observed without clear sex-dependent differences (Ghezellou et al., 2024). **Figure 2D** summarizes the regulatory mechanisms affecting hepatic carbohydrate and lipid metabolism during *S. mansoni* infection from our research and published studies. The direction of regulation of key enzymes involved in hepatic metabolism of bisex-infected animals or hepatocytes *in vitro* stimulated with egg-derived antigens (SEA) is detailed in **Table 1**. Increasing evidence indicates that mitochondrial phenotypes differ between males and females in the liver, with important implications for inflammatory susceptibility and disease progression. Female hepatic cells generally exhibit a more oxidative, OXPHOS-oriented mitochondrial metabolism characterized by greater respiratory efficiency and enhanced resistance to oxidative stress. In contrast, male liver cells appear more prone to inflammation-associated metabolic reprogramming toward glycolysis, particularly under conditions of chronic immune activation (Chen J. et al., 2025). These sex-specific differences are thought to be driven, at least in part, by hormone-dependent regulation of mitochondrial function and antioxidant capacity within the hepatic microenvironment. Such divergent mitochondrial phenotypes may contribute to the heightened inflammatory tone observed in male livers and thereby increase susceptibility to progressive liver inflammation, fibrosis, and subsequent disease severity (Nadal-Casellas et al., 2010).

**Table 1:** Regulation of key enzymes and factors involved in hepatic metabolism after infection with *S. mansoni* or incubation with *S. mansoni* egg-derived antigens.

Metabolic pathway	Proteins and factors	Function	Host gender		*In vitro*
			♂	♀	
Carbohydrate metabolism	PKM1	Glycolysis	↑	↑	↑
	GS	Glycogen synthesis	↓	↓	↓
	G6PD	Pentose phosphate pathway	↑	↑	↑
	PCK2	Gluconeogenesis	↔	↔	
Lipid metabolism	FAS	Fatty acid synthesis	↓	↓	↓
	ACC1		↓	↓	↓
Oxidative stress	ROS (Bülow et al., 2023; Novaes et al., 2025)	Oxidative stress	↔	↑	↑

The direction of regulation of key enzymes involved in hepatic metabolism is shown for *S. mansoni*-infected animals (or SEA-stimulated hepatocytes *in vitro*), relative to at least one control group. ↑, upregulation; ↓, downregulation; ↔, no significant regulation; ♂, liver lysates of male hamsters; ♀, liver lysates of female hamsters. Results include data from the literature and own data in mice and cell culture.

Male ApoE^−/−^ mice are widely used as a model of dysregulated lipid metabolism and chronic inflammation, providing a valuable tool to study the interplay between metabolic imbalance, immune activation, and liver pathology (Lo Sasso et al., 2016). Schistosomiasis has been shown to protect male ApoE^−/−^ mice from the development of atherosclerosis, diabetes and obesity while altering the metabolism of liver macrophages. In the study mentioned the authors found that macrophages generated from bone marrow myeloid progenitors from infected male mice have long-lived increases in their basal metabolism of lipids. Their results suggest that metabolic reprogramming is long-lasting without exposure to active infection, and develops a memory-like phenotype in myeloid cells. This reprogramming was transferrable via bone marrow transplantation. Under nutrient deprivation, hepatocytes shift their metabolism into a survival mode: glucose is supplied via glycogenolysis and gluconeogenesis, lipid stores are increasingly oxidized, and amino acids are mobilized for energy production (Rui, 2014). Concurrently, energy-consuming processes are downregulated, autophagy is activated, and antioxidant defenses are mobilized to preserve cellular integrity (Byrnes et al., 2022). Novaes et al. recently demonstrated that progressive liver fibrosis, sustained high levels of reactive oxygen species (ROS), pronounced DNA damage, and a reduction in p16 and p21 expression are associated with impaired hepatocyte proliferation during the chronic phase of *S. mansoni* infection. Consequently, pharmacological inhibition of both the infection and granulomatous inflammation is crucial to prevent these indicators of premature senescence linked to hepatocyte replication deficits and to promote liver regeneration in *Schistosoma mansoni* infection (Novaes et al., 2025).

Amer et al. also pointed out that schistosome infection in mouse models leads to an increase in Th2 and regulatory T cell cytokines (Amer et al., 2023). In addition, changes in the lipid profile and blood sugar levels were observed in infected diabetic and obese animals. Surprisingly, *schistosome* infection had a positive effect on insulin levels in obese mice. T2DM and obesity, however, increased tissue egg counts and fibrosis, while the infection enhanced lipid profiles and insulin sensitivity in obese mice (Amer et al., 2023). These conditions contribute to a specific hepatic microenvironment which affects mitochondrial function. Mitochondria are energy producers in cells and are responsible for maintaining normal functions by controlling mitochondrial redox homeostasis, metabolism, bioenergetics, and cell death pathways (Chen T.-H. et al., 2025).

### Sex-dependent autophagy in schistosomiasis

As the liver undergoes metabolic reprogramming during schistosomiasis, autophagy—a key catabolic process involved in degrading and recycling damaged cellular components—has emerged as an important mechanism in maintaining hepatic homeostasis and regulating oxidative stress (Rothermel and Diwan, 2021). Autophagy may play a dual role in liver pathology during *Schistosoma* infection: promoting cellular survival in response to inflammation and oxidative damage, or contributing to disease progression under persistent stress conditions.

While some studies have examined autophagic responses during infection, there is limited and inconsistent evidence regarding sex-specific regulation of autophagy in the host. Importantly, most studies have not been explicitly designed to assess sex-dependent effects, and comparisons across studies are complicated by substantial methodological heterogeneity, including differences in parasite species (*S. mansoni vs. S. japonicum*), host mouse strains, infection duration, infection methods, and endpoints analysed (Chen et al., 2015; Zhang et al., 2022; Härle et al., 2023; Hasby Saad et al., 2024; Yu et al., 2024; Yuan et al., 2024). For instance, a study using *S. japonicum*-infected female mice reported a significant reduction in liver autophagy markers (Beclin1, p62, LC3BII/I, Atg7, Atg12) at two to six weeks post-infection, correlating with hepatic injury and fibrosis development (Chen et al., 2015). However, the absence of a male control group limits any conclusions about sex-specific regulation. Similarly, *in vitro* experiments using schistosomal egg antigens (SEA) showed decreased expression of autophagy-related proteins in hepatic macrophages (Zhu et al., 2018). While informative, such *in vitro* models lack systemic hormonal influences and cannot be used to assess sex differences. In contrast, other studies demonstrated autophagy activation in male mice following *S. japonicum* infection or its suppression under hepatocyte-specific FXR deficiency (Zhang et al., 2022), suggesting the possibility of sex- and receptor-dependent modulation (Dean et al., 2021; Patton et al., 2024). However, these findings remain preliminary and often rely on differing mouse backgrounds (e.g., C57BL/6, BALB/c, Swiss albino), which may further influence immune and metabolic responses. **Table 2** summarizes selected studies reporting on hepatic autophagy in murine schistosomiasis models. The inconsistent results and absence of well-controlled comparative studies highlight the need for future research directly addressing the role of sex in hepatic autophagy during *S. mansoni* infection.

**Table 2:** Overview of studies examining autophagy regulation in murine schistosomiasis, annotated by host sex, parasite species, and study design.

Ref.	Results	Host sex	Mouse strain	Parasite species	Model
(Chen et al., 2015)	*S. mansoni* ➔; hepatic mitophagy ↓	♂	BALB/c	*S. mansoni*	*in vivo*
(Yu et al., 2024)	hepatic autophagy and apoptosis ↓	♀	C57BL/6	*S. japonicum*	*in vivo*
(Yuan et al., 2024)	Hepatic autophagy in progenitor cells ➔; miRNAs inhibit fibrosis	♂	C57BL/6	*S. japonicum*	*in vivo*
(Härle et al., 2023)	c-Jun signaling not involved in regulating hepatic autophagy	♂	C57BL/6	*S. mansoni*	*in vivo*
(Zhang et al., 2022)	FXRΔHep➔; autophagy ↓ & liver injury ↑	♂	C57BL/6	*S. japonicum*	*in vivo*
(Hasby Saad et al., 2024)	Chloroquine ➔; inflammatory markers↑ & PZQ effect ↑	♂	Swiss albino	*S. mansoni*	*in vivo*

Due to heterogeneity in models and incomplete sex-specific analyses, these studies should be interpreted with caution.

Finally, chloroquine—an inhibitor of autophagosome–lysosome fusion—has been tested in *S. mansoni*-infected male mice, where it enhanced the schistosomicidal efficacy of praziquantel and modulated inflammatory signaling (Hasby Saad et al., 2024). Although promising, such interventions have not been evaluated in both sexes, limiting conclusions about differential efficacy or toxicity. Moving forward, studies systematically controlling for host sex are critical to disentangle the role of autophagy in disease progression and therapy response during schistosomiasis.

### Sex-specific oxidative stress and autophagy in *S. mansoni* infection

**Figure 3:**
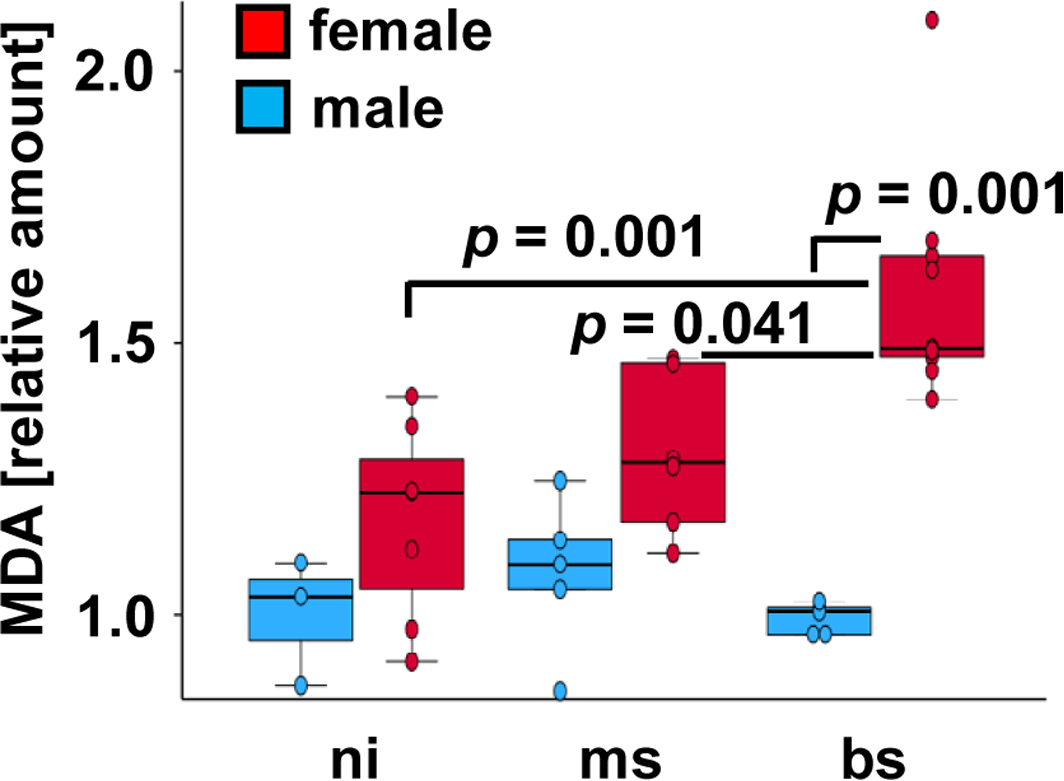
Comparison of hepatic MDA levels in female and male hamsters infected with *S. mansoni*. MDA levels were normalized by calculating the ratio to the mean value of the control group on the respective test day. Statistical differences were assessed using the Kruskal-Wallis test followed by pairwise group comparisons with Bonferroni correction. Data represent mean values from at least three technical replicates (Bülow et al., 2023). ni non-infected, n=3 (male) and n=7 (female); ms monosex, n=5 (male) n=6 (female); bs bisex, n=5 (male) n=10 (female).

Oxidative stress, closely related to autophagy, plays a detrimental role in liver damage and fibrosis during *S. mansoni* infection. ROS directly affects autophagy via several mechanisms, including S-glutathionylation at AMPK and oxidation of autophagy-related protein 4 (ATG4) (Scherz-Shouval et al., 2007). However, autophagy and oxidative stress are also indirectly linked via p62 and Keap1/Nrf2, resulting in the transcription of antioxidant genes (Tang et al., 2019). In female hamsters, *S. mansoni* infection induced metabolic exhaustion and a significant redox imbalance in the liver. This led to parenchymal damage, which may increase the susceptibility to develop cancer (Roderfeld et al., 2020; Bülow et al., 2023). Recently, metabolic reprogramming by *S. mansoni* was shown to cause oxidative stress in hepatocytes, leading to DNA damage and hepatocellular proliferation in female hamsters (Bülow et al., 2024). *S. mansoni* egg-induced oxidative stress triggered a replication stress response (Bülow et al., 2024). Under these conditions, MCM replication licensing proteins were upregulated in both, male and female hamsters (Bülow et al., 2024). Upregulation of MCM proteins might have multiple biological consequences, particularly with respect to cell division and genomic stability. Elevated MCM expression is frequently observed in various tumors, where it correlates with heightened proliferative capacity due to the intensive utilization of the DNA replication machinery (Yu et al., 2020). These harmful effects of oxidative stress might be mitigated by the administration of reactive oxygen-species scavengers such as reduced glutathione (Bülow et al., 2024). The oxidative stress marker malondialdehyde (MDA) was elevated in the livers of *S. mansoni*-infected female hamsters, however, not in male hamsters (**Figure 3**) (Bülow et al., 2023). Compared to the controls, however, no increase of the MDA level in the liver of infected male hamsters was observed (**Figure 3**). Notably, liver proteome analysis revealed a significant upregulation of hepatocellular metallothionein 2 (MT2) in infected males, but not in females (Ghezellou et al., 2024). MT2 is essential for heavy metal detoxification, oxidative stress defense, and metabolic regulation via zinc donation (Cuillel et al., 2014). Its induction during *S. mansoni* infection may serve as a protective response to oxidative stress caused by egg antigen products. These results suggest that male hamsters may have a more effective counter-regulatory mechanism against oxidative stress than their female counterparts. Relevant data were published recently (Al-Gubory and Garrel, 2016). Female liver has indeed a greater superoxide dismutase (SOD2) activity than male liver. Male liver has greater glutathione reductase activity than female liver. In addition male liver has a higher total GSH and GSSG content than female liver (Al-Gubory and Garrel, 2016).

On the other hand there is substantial evidence that females exhibit superior protection against reactive oxygen species (ROS) compared to males, contributing to increased lifespan and a lower incidence of most chronic diseases. The overview consolidates the most recent findings on sex-specific differences in the regulation of redox homeostasis (Tiberi et al., 2023).

Reactive oxygen species (ROS) are upstream modulators of autophagy and act at several levels (Filomeni et al., 2015). One explanation for this regulation is that nutrient deprivation leads to energetic stress, which causes mitochondrial overload, resulting in increased electron loss and ROS production (Filomeni et al., 2015). Sex-specific differences in oxidative stress diseases are common, with females usually being more resistant to cell death caused by oxidative stress (Tower et al., 2020). Positive effects of estrogen, improved mitochondrial function, and increased expression of stress-response genes in female cells might be the reason (Mahalingaiah et al., 2015). The response of female and male organisms to oxidative stress is highly complex and multifactorial (Tower et al., 2020). Several key genetic and physiological characteristics confer a relative advantage to females with respect to resilience against oxidative and proteotoxic stress, including glucose-6-phosphate dehydrogenase (G6PD), heat shock proteins (HSPs), and numerous additional protective mechanisms (Tower et al., 2020). Beyond these factors, immunological components may also contribute to sex-specific differences and could be linked to the greater propensity of females to develop maladaptive immune responses. Consequently, the etiology of the elevated malondialdehyde (MDA) levels observed in the hepatic tissue of female animals (**Figure 3**) remains insufficiently understood. Importantly, MDA represents an indicator - but not definitive evidence - of oxidative stress, as increased levels may also arise from inflammatory processes, dietary influences, or alterations in lipid metabolism.

### Translational applicability

Although most insights into sex-specific hepatic responses during *S. mansoni* infection are derived from experimental models, these findings may offer preliminary clues relevant to human schistosomiasis. Differences in liver metabolism, immune modulation, and hormone signaling between male and female hosts could contribute to variation in disease progression and treatment responses. However, the transferability of these observations to humans remains uncertain due to interspecies differences and the controlled nature of experimental infections.

Sex-based metabolic variation—such as in lipid metabolism, oxidative stress response, or amino acid turnover—may influence liver pathology and could, in principle, affect pharmacokinetics or therapeutic outcomes, including responses to praziquantel. While there is currently limited clinical evidence directly linking these factors, the potential for sex-specific differences in drug metabolism or susceptibility to liver fibrosis warrants further investigation in human cohorts.

Moreover, identifying metabolic signatures that differ by sex in animal models could help generate hypotheses for future clinical studies aimed at developing non-invasive biomarkers of disease severity. Such efforts may eventually support more individualized approaches to disease monitoring and management.

In light of these considerations, we emphasize the importance of designing future preclinical and clinical studies that intentionally incorporate sex as a biological variable. This will be essential to determine whether the sex-specific metabolic differences observed in animal models hold relevance for human schistosomiasis and could inform treatment strategies in an evidence-based manner.

### Limitations

This article is subject to several limitations stemming from the heterogeneity of the studies included. First, the analyzed data originate from experiments involving different *Schistosoma* species, primarily *S. mansoni* and *S. japonicum*, which differ in egg production rates, tissue tropism, and immunopathological outcomes. These biological differences may influence host metabolic responses in a species-specific manner. Second, there is considerable variability in experimental design across studies. Key differences include the age of the host at the time of infection, the number of cercariae used for infection, the infection method (e.g., percutaneous vs. intraperitoneal or intravenous injection), and the duration of infection before sample collection. These factors can significantly influence disease progression and host response, complicating direct comparisons. Furthermore, different mouse strains used across studies, each with distinct genetic backgrounds that affect immune responses and metabolic pathways. The use of various host sexes, often without sex being a primary experimental variable, further complicates interpretation. In many cases, male- or female-only host data were included based on study availability rather than intentional sex-based design. While efforts were made to critically assess these differences and interpret findings within their respective contexts, the variability across models and methodologies must be considered when drawing broader conclusions. These limitations highlight the need for more standardized and sex-inclusive experimental designs to better understand host-specific metabolic responses to *Schistosoma* infection. While mouse models provide valuable insights into the pathophysiology of *Schistosoma* infection, the transferability of findings to humans remains limited due to fundamental interspecies differences in immune responses, liver metabolism, and disease progression. The rodent models are well-established for *S. mansoni* research, and notably, most biochemical effects observed in *S. japonicum*-infected hamsters align with those previously reported in mice. While schistosomiasis in humans shows a male-sex bias, female mice infected with S*. mansoni* exhibited a higher parasite burden and stronger proinflammatory Th1 responses than males. Conversely, human male hosts are more prone to severe hepatic fibrosis compared to females (Silva-Teixeira et al., 2004). Additionally, hepatic *S. mansoni* egg accumulation has been shown to inversely correlate with the Th2 immune response (Russ et al., 2024). This partial reversal of sex differences complicates the interpretation of mouse model data in relation to human schistosomiasis (Bernin and Lotter, 2014). Additionally, autophagy regulation varies by sex and by age, another important key factor (Müller et al., 2024). In this context, however, many studies failed to report or consider the age of infected animals.

In summary, the results obtained in the reviewed studies, including our data, suggest that *S. mansoni* infection might reprogram hepatic carbohydrate and lipid metabolism in a sex-independent manner. In contrast, oxidative stress regulation differs by gender, with males managing oxidative stress more effectively than females. Literature data also indicate a sex-specific regulation of autophagy. Therefore, schistosomiasis-associated hepatic autophagy and its inhibition by chloroquine may offer a novel option for therapeutic strategies of patients.

To address the many unanswered questions in this emerging field of sex-related disease effects, further research is urgently needed—particularly studies that investigate the molecular mechanisms underlying these differences in greater detail. Such efforts will enhance our understanding of sex-specific disease manifestations and may ultimately guide the development of more targeted, and potentially sex-tailored, therapeutic strategies for schistosomiasis.

## Data Availability

The original contributions presented in the study are included in the article/Supplementary Material. Further inquiries can be directed to the corresponding author.
